# Access to primary and specialized somatic health care for persons with severe mental illness: a qualitative study of perceived barriers and facilitators in Swedish health care

**DOI:** 10.1186/s12875-017-0687-0

**Published:** 2018-01-09

**Authors:** Elisabeth Björk Brämberg, Jarl Torgerson, Anna Norman Kjellström, Peder Welin, Marie Rusner

**Affiliations:** 1 Unit of Intervention and Implementation Research for Worker Health, Institute of Environmental Medicine, Karolinska institutet, 171 77 Stockholm, Sweden; 2 Närhälsan Eriksberg Primary Health Care Centre, Sjöporten 4, 417 64 Göteborg, Sweden; 3 Department of Psychosis, Sahlgrenska University Hospital, 431 80 Mölndal, Sweden; 4 Department of Data Management and Analysis, Head Office, Region Västra Götaland, 541 80 Skövde, Sweden; 5 Centre for Equity in Health, Region Västra Götaland, Regionens Hus, 405 44 Göteborg, Sweden; 6 Department of Research, Södra Älvsborgs Hospital, Brämhultsvägen 52, 501 82 Borås, Sweden; 7 Institute of Health and Care Sciences, Sahlgrenska Academy, University of Gothenburg, 405 30 Gothenburg, Sweden

**Keywords:** Barriers and facilitators, Health services accessibility, Qualitative research, Semi-structured interviews, Severe mental illness

## Abstract

**Background:**

Persons with severe mental illness (e.g. schizophrenia, bipolar disorder) have a high prevalence of somatic conditions compared to the general population. Mortality data in the Nordic countries reveal that these persons die 15–20 years earlier than the general population. Some factors explaining this high prevalence may be related to the individuals in question; others arise from the health care system’s difficulty in offering somatic health care to these patient groups. The aim of the present study was therefore to explore the experiences and views of patients, relatives and clinicians regarding individual and organizational factors which facilitate or hinder access to somatic health care for persons with severe mental illness.

**Methods:**

Flexible qualitative design. Data was collected by means of semi-structured individual interviews with patients with severe mental illness, relatives and clinicians representing primary and specialized health care. In all, 50 participants participated.

**Results:**

The main barrier to accessing somatic care is the gap between the organization of the health care system and the patients’ individual health care needs. This is observed at both individual and organizational level. The health care system seems unable to support patients with severe mental illness and their psychiatric-somatic comorbidity. The main facilitators are the links between severe mental illness patients and medical departments. These links take the form of functions (i.e. systems which ensure that patients receive regular reminders), or persons (i.e. professional contacts who facilitate patients’ access the health care).

**Conclusions:**

Health care services for patients with severe mental illness need reorganization. Organizational structures and systems that facilitate cooperation between different departments must be put in place, along with training for health care professionals about somatic disease among psychiatric patients. The links between individual and organizational levels could be strengthened by introducing professional contacts, such as liaison physicians and case managers. This is also important to reduce stress and responsibility among relatives.

**Electronic supplementary material:**

The online version of this article (10.1186/s12875-017-0687-0) contains supplementary material, which is available to authorized users.

## Background

Persons with severe mental illness (SMI), defined as schizophrenia, bipolar disorder and psychosis, have a high prevalence of somatic medical conditions compared to the general population [1]. Mortality data for persons with SMI in the Nordic countries (Denmark, Finland, Sweden) reveal that these persons die 15–20 years earlier compared to the general population [2], mainly because of cardiovascular disease and cancer [3]. Persons with SMI experience a high number of risk factors that increase the prevalence of cardiovascular diseases and diabetes mellitus [4]. This is related to the side effects of prescribed antipsychotic medications [5], suboptimal lifestyle, lack of physical activity [3], poor diet and high rates of smoking [6–8]. A recent report from the Västra Götaland region of Sweden confirms the international data on mortality rates and the prevalence of somatic diseases within this patient group [9].

Other factors that explain the high prevalence of somatic diseases among persons with SMI are related to the health care system’s difficulty in providing somatic health care which is appropriate to these persons’ health care needs. Previous studies have explored factors which are barriers to and facilitators of access to health care for persons with psychiatric-somatic comorbidity [10–12]. Barriers at an organizational level were lack of communication and poor coordination between mental health and somatic care [11]. Lack of resources and productivity pressure, untrained staff, insufficient clinical support for staff, lack of established routines for referral of patients and lack of inter-agency collaboration were also seen as barriers [10, 12, 13]. Patient-related barriers were mental health stigma [14], along with socioeconomic barriers such as poverty, insecure housing and lack of transportation [15]. Difficulty in establishing and maintaining contact with a general practitioner also hindered access to health care [15]. Organization-related facilitators of access to health care were interdisciplinary, collaborative models of primary health care provision, which kept patients in regular contact with a primary health care clinician [15].

A limitation of previous research into barriers and facilitators is that it mostly uses data collected in countries other than Sweden, with different health care systems and treatment strategies for patients with SMI. These studies often lack a theoretical basis for identifying barriers and facilitators. We have therefore conducted a qualitative study of SMI patients, their relatives and clinicians working in primary and specialized health care. The study evaluates the factors which according to SMI patients, their relatives and clinicians, influence patient access to somatic health care. We aim to identify factors that may either hinder or facilitate implementation. To this end we will use the consolidated framework for implementation research (CFIR) [16], which will form the theoretical basis for the present study. According to CFIR, barriers or facilitators can be influenced by factors related to the following domains: intervention characteristics; characteristics of individuals; and inner and outer settings. Given the magnitude of the individual and societal costs related to the psychiatric-somatic comorbidity and disease burden, we need to gain a deeper understanding of the barriers and facilitators which affect patient access to health care in Sweden.

## Aim

The aim was to explore the experiences and views of patients, relatives and clinicians regarding individual and organizational factors which facilitate or hinder access to somatic health care for persons with SMI.

## Methods

### Design

The present study applies a flexible, qualitative design. Qualitative data was collected by means of semi-structured individual interviews with patients with SMI, relatives and clinicians representing primary and specialized (somatic in-patient and psychiatric out-patient) health care. The qualitative interviews are based on the assumption that the interaction between the interviewer and the participant is an interplay between the interviewer’s awareness of what and how the participant presents his/her answers and the participant’s ability to explore and clarify his/her individual experiences and thoughts [17]. The method is reported in line with Tong A, Sainsbury P and Craig J [18].

#### Setting and participants

In Sweden, responsibility for health care is shared by national government, 20 county councils and 290 municipalities. The system is mainly tax funded. The government sets the political agenda and the principles and guidelines for the health care system by means of national legislation and/or agreements with the county councils and municipalities. Health care (primary as well as specialized care) is provided and funded chiefly by the county councils. The municipalities are responsible for the care and social security of people with psychiatric disorders [19]. As a consequence of this division of responsibilities, patients with SMI may have several health care contacts and providers.

The present study was conducted in a region of western Sweden. The selection of participants for the interviews was guided by strategic sampling in order to get a variation in participant experience of the questions under study. The study population consisted of persons suffering from SMI (hereafter called patients), relatives and clinicians. The recruitment of patients was based on psychiatric diagnosis, age, gender, and whether they lived in an urban or rural area. The inclusion criteria were: ongoing contact with psychiatric out-patient care; psychiatric diagnosis of bipolar disorder type I or II, psychosis or schizophrenia (ICD-10 codes: F2, F30.2, F31.2, F31.5, F32.3, F33.3); contact during the previous 12 months with primary or specialized health care for a somatic disease, such as type 2 diabetes mellitus, heart failure, hypertension or cancer. Exclusion criteria were: in-patient psychiatric care during the previous 12 months; ongoing investigation of patient’s complaints by the patients’ advisory committee. The selection of relatives was based on type of relationship (i.e. parent, adult child, sibling, spouse, co-habitant); the patient’s psychiatric diagnosis and the patient’s somatic diagnosis. The clinicians were chosen to represent different fields of primary and specialized health care (primary, somatic in-patient care, psychiatric out-patient care).

We presented the aims of the study to five managers representing five psychiatric out-patient units, 23 managers representing 23 units in primary and specialized care and six advocacy groups working with bipolar disorder or schizophrenia. All received oral and written information. The managers were asked to recruit clinicians and patients (primary and specialized health care). The representatives of the advocacy groups were asked to recruit relatives of SMI patients.

Four psychiatric out-patient units, 10 primary and specialized health care units and six of the advocacy groups helped to recruit patients, clinicians and relatives. The recruitment process was performed by unit managers (who recruited clinicians and patients) and representatives of advocacy groups (who recruited relatives). They received oral and written information about the recruitment process from the research group. The process resulted in 18 patients, 17 relatives and 21 clinicians agreeing to participate. Because the recruitment was performed by persons outside the research group, we cannot give an exact number for how many persons were invited to participate. Of those who initially agreed to participate, four of the patients and two of the relatives withdrew later for personal reasons. None of the clinicians withdrew from the study. Thus, interviews were conducted with 14 patients, 15 relatives and 21 clinicians. All participants received written information regarding the study’s aim and content. They were given the choice of having the interview in their home town or nearby, at a time they found convenient.

#### Data collection

The interviews were held between May and September 2016. All interviews were semi-structured, i.e. three interview guides comprising a pre-determined set of open-ended questions were used. The interview guides were constructed for patients, relatives and clinicians and are further described below. All the interview guides were reviewed by the research group and pilot-tested at the first interview. Only small changes were made, such as a minor rephrasing of questions or changing the order of the questions. All interviews were conducted at face-to-face meetings, with no one other than the interviewer and the participant present. All except one were digitally recorded and transcribed verbatim. In the case of the latter interview, the patient did not allow the interview to be recorded and therefore notes were taken. No repeat interviews were carried out. A description of the participants is presented in Table 1.

**Table 1:** Description of participants

Patients *N* = 14		
Female/male	10/4	
Age, years (mean)	35–77 (54)	
Psychiatric diagnosis (self-reported)		
*Bipolar disorder*	8	
*Schizophrenia/psychosis*	6	
Relatives *N* = 15		
Female/male	13/2	
Age, years (mean)	33–80 (55)	
Type of relationship		
*Adult child*	1	
*Married*	3	
*Parent*	6	
*Sibling*	5	
Psychiatric diagnosis (self-reported by the relatives)		
*Bipolar disorder*	4	
*Schizophrenia/psychosis*	11	
Clinicians *N* = 21		
Female/male	17/4	
Profession		Workplace
*Assistant nurse*	3	3 Psychiatric out-patient care
*Behavioral scientist*	2	2 Psychiatric out-patient care
*Physician*	7	3 Primary health care 1 Psychiatric out-patient care 3 Somatic in-patient care
*Physiotherapist*	1	1 Psychiatric out-patient care
*Registered nurse*	8	4 Primary health care 2 Psychiatric out-patient care 2 Somatic in-patient care

The interview guides were developed for this study with inspiration from the CFIR guide http://www.cfirguide.org/. The following domains and their constructs were addressed: outer setting (patient needs and resources); inner setting (structural characteristics, access to knowledge and information, leadership engagement); individual characteristics (knowledge and beliefs). The guide for patients consisted of eight questions, starting with “Can you tell me about when you had to contact the health services because of a physical illness?”. The guide for the relatives consisted of seven questions and started with: “Can you tell me about a situation in which your relative with SMI had to contact the health services because of a physical illness?”. The guide developed for clinicians contained 14 questions and started with: “Can you tell me about how your unit meets the somatic health care needs of patients with SMI?”. The interview guides are presented in the Additional file 1. Follow-up questions (for example “Could you please tell me more about that situation?”) were asked in all interviews when necessary.

Fourteen patients were interviewed in face-to-face meetings by the first author (EBB). She has a PhD, is an assistant professor and has broad experience of and training in qualitative methodology. The interviews lasted between 30 and 90 min and took place in public places (such as libraries or cafés) or in the interviewee’s home. The patients had sought help from the primary or specialized care services for asthma, diabetes, various types of cancer, hypertension, myocardial infarction, renal insufficiency and non-specific musculoskeletal pain. All patients reported having more than one somatic diagnosis. Diabetes was the most common diagnosis. Fifteen relatives were interviewed in face-to-face meetings by a research assistant (SE). The interviews lasted between 15 and 50 min and were conducted in the interviewee’s home, at the advocacy group or in a public place. Twenty-one clinicians were interviewed in face-to-face meetings by a research assistant (AJ). The interviews lasted between 10 and 45 min and were conducted at the clinicians’ workplaces. Both research assistants were women and trained nurses. One has a PhD and the other a MSc. Both had prior training in qualitative methods and interview techniques.

### Data analysis

The data (recordings and transcripts) were cross-checked for accuracy by ANK and JT. The data was analyzed by means of qualitative content analysis [20]. The analysis was performed according to the following steps: 1) The transcribed interviews were read through several times to gain an overall insight into the content. 2) All transcripts were explored by open coding, by marking sentences and/or paragraphs related to the study’s aim and then condensed into meaning units, i.e. the marked text was summarized. 3) The meaning units were labeled with codes, which reflected different aspects of the content. 4) All meaning units and codes were transferred from the text to coding sheets in a MS Word document. 5) The codes were compared in order to identify similarities and differences. 6) Codes with similar content were classified as one category. 7) All codes and categories were compared with the interviews to ensure that they were appropriately linked to each other. 8) The categories were designated as barriers or facilitators according to their content. 9) Themes describing main barriers and facilitators were derived from the respective categories. Examples of the data analysis process are given in Table 2. Throughout the analysis procedure, the authors went back and forth between the transcripts, meaning units, codes and categories to make sure that the results had been conceptually created and developed from the data by the analysts and did not lose their content in relation to the context described in the interviews. EBB had chief responsibility for the analysis in close collaboration with MR. In the latter phase of the analysis, the authors jointly discussed the analysis and agreed on the results. Short quotations from the data were used to illustrate the link between the data and the analysis.

**Table 2:** Description of the analysis process

Meaning unit	Code	Category	Barrier/facilitator at individual or organizational level	Theme
When I finally did go to emergency department for help, my blood sugar levels were so high that I really shouldn’t have been able to stay upright. What I mean is, the reason why I waited so long before getting help was that I’m afraid they’re going to tell me you’ve got these problems because of your bipolar illness and the side effects of your drugs. You’re afraid to go to the doctor’s because that’s the last thing you want is to be told.	Afraid of not being taken seriously	Self-stigmatization	Individual-level barrier	Main barrier: the gap between the organization of the health care system and patients’ health care needs.
When our patients seek treatment for their somatic conditions it’s usually got quite advanced. They don’t seek help for the early symptoms and they often aren’t already registered at a health centre. If you need help, it’s very difficult for them to make a phone call, wait in a telephone queue, wait to be phoned back. Which means that I, as the person who is treating them, often make the phone calls for my patients to make things easier.	Making it easier for patients to get in touch with the health services.	Access to a professional contact	Organizational-level facilitator	The main facilitators: the links between the patients with SMI and the health care departments.

## Results

For an overview of the results, see Table 3.

**Table 3:** Overview of the findings

	Individual-level	Organizational-level	Themes
Barriers	Self-stigmatization^a^	The fragmentation of the previously comprehensive county council primary care system^a, b, c^	The main barrier is the gap between the organization of the health care system and the patients’ health care needs
	The patient’s cognitive disability^b, c^	The lack of cooperation between different parts of the health care system^a, b, c^	
	The clinician’s lack of knowledge of mental illness^c^	Lack of psychiatric expertise^b, c^	
		Single-disease paradigm^b, c^	
Facilitators	The relative as a spokesperson^b^	Access to a professional contact person^a, b, c^	The main facilitators are the links between the patients with SMI and the health care departments
	The clinician’s own interest in developing a better understanding^c^	Continuity among clinicians^a, b, c^	
		Annual reminders^a, c^	

^a^Barrier/facilitator reported by patients

^b^Barrier/facilitator reported by relatives

^c^Barrier/facilitator reported by clinicians

## Barriers

The main barrier is the gap between the organization of the health care system and the patients’ health care needs. This is observed at both individual and organizational levels. In particular, the somatic health care system seems unable to support SMI patients with psychiatric-somatic comorbidity. The data indicates that the system relies on the individual’s ability to initiate contact with health care providers, which is a problem for these patients. Another barrier is the lack of contact between different health care sectors.

### Individual-level barriers

The results indicate that patients’ self-stigmatization and cognitive disabilities together with clinicians’ lack of knowledge of SMI are individual factors which constitute barriers to obtaining somatic health care.

All patients reported **self-stigmatization** as a barrier to accessing health care. They described previous experience of not being believed and not being taken seriously. This, in combination with their own thoughts about psychiatric disease, sometimes made the patient doubt whether the somatic symptoms were “for real”. Previous experience of seeking help had left patients with the impression that mental illness was used as a framework for interpreting their somatic symptoms. The perceived tendency of clinicians to see the psychiatric diagnosis first and foremost was a major concern for the patients and seemed to increase their self-stigmatization.

*When I came in they couldn’t even do tests at the emergency clinic. So it was a real emergency. But what I mean, what I want to get to, is that the reason I just hung around at home so long is that I’m scared they’re going to say “You’ve got these problems because of your bipolar diagnosis and the side effects of your medication.” You’re scared of ever going to the doctor’s, because that’s absolutely not what you want thrown at you. (Patient 2).*

*It’s a lot like you’re imagining things. Because you think… you’ve got this terrible thing, and it becomes a kind of self-condemnation. That it’s something bad. And then you kind of end up at the back of the queue. You think you’re being discriminated against all the time. And maybe that’s not how things are in reality always. But you think, as soon as they get to know about this (the psychiatric diagnosis, author’s note) I’ll get worse treatment. Because I’m not worth as much in society. If you don’t pay tax and are ill. It’s terrible. (Patient 13).*

Relatives and clinicians reported that a **patient’s cognitive disabilities** were barriers to accessing health care. Relatives had first-hand experience of how patients’ disabilities could affect medical visits, with clinicians interpreting symptoms as resulting from the psychiatric condition rather than as symptoms of a somatic disease. As one parent said at the interview:

*Now, I haven’t been with her every time, but when she talks about it she feels that they just… That they’ve largely neglected her, and that’s how I see it too, even when I phone and talk to them I feel that… it’s as if she’s been labelled. She does behave a bit differently. She has a different way of talking, it might be that kind of thing that makes people not believe her. (Relative 10)*

This barrier was also mentioned by the clinicians working in somatic health care. They sometimes found it hard to understand psychotic patients’ descriptions of the symptoms they were experiencing. As a consequence, the clinicians struggled to get the patient’s medical history, sometimes with the help of other persons such as family members. The clinicians themselves suspected that they often underestimated patients’ symptoms because of the difficulty of establishing the medical history.

*I can see extra needs, these particular patients can be more difficult to assess and in some cases can be dissimulating, that they don’t express the morbidity they may have in the same way other people do. There’s a risk that you don’t take their complaints as seriously, I think, there’s a risk of making light of them in some way. I worked in psychiatric care for a while before I became a doctor. That’s why I’m extra cautious in a way. (Clinician 1 somatic in-patient care).*

**The clinicians’ lack of knowledge of mental illness** was a barrier reported by the clinicians working in somatic health care (primary and specialized in-patient care). Their medical training had included psychiatry, but this was perceived as insufficient. The clinicians also recognized that they had difficulty in dealing with non-adherence to medication and/or treatment in this patient group. They identified the need for training and cooperation to address these problems.

*Maybe you don’t have good enough specialist knowledge about these groups (author: patients) and the problems they can have, what we do in principle is treat them like any other patient. Then you usually get, can get problems with communication, or compliance, and then we usually get help from a psychiatric consultant. (Clinician 15 somatic in-patient care).*

### Organizational-level barriers

The results indicate that, according to the interviewed patients, relatives and clinicians, there are a number of organizational factors that negatively impact the patients’ ability to access health care.

**The fragmentation of the previously comprehensive county council primary care system** was described as a barrier by patients, relatives and clinicians. Since 2009 patients have had the right to choose their primary care provider. Private care providers and free-market competition between these providers was also introduced. The clinicians in the present study regarded this system as a hindrance because it does not give priority to clinical continuity and gives no incentives for clinicians at different units to cooperate with each other.

*Previously (author: before the introduction of the right to choose) you never talked about medical priorities in relation to what the diagnosis was and what you got money for. But today everything is steered by which patients and diagnoses are registered at the health centre. You should really… If you haven’t got enough time for everything, you should remove what you don’t get money for. We don’t actually say that, but that’s the reality. (Clinician 17 primary health care).*

**The lack of cooperation between different parts of the health care system**, i.e. between primary and specialized care, within specialized institutions and between the county council’s health services and municipal care, was seen as a barrier by patients, relatives and clinicians. The patients felt that, in one way, the lack of cooperation protected their integrity, such as when the health care institutions did not exchange patient-related information. On the other hand, this put the onus on the patients themselves to obtain information and decide what the next step should be.

*So I go to the bipolar clinic, you know that, and then I get sent to the emergency unit, and at the emergency unit they say no, you’re heart’s beating slowly because of your medication. So I go back to the bipolar clinic and “No, I don’t think so because the dose is so low”. Ok. You can never get the whole picture, it’s always just bits like this, so you just have to try to piece it together yourself. (Patient 10).*

The lack of cooperation was seen by the clinicians and relatives as reflecting the way in which specialized care focuses on a **single-disease paradigm**. This paradigm was seen as a facilitator for highly specialized care, but also as a barrier for persons with psychiatric-somatic multi-morbidity with regard to cooperation between different care sectors such as psychiatry and medicine, and primary and specialized care.

Almost all of the clinicians and relatives highlighted the **lack of psychiatric expertise** (i.e. clinicians such as physicians, psychiatrists, registered nurses with a specialization in psychiatry) as a barrier to accessing somatic health care. The clinicians felt they had limited opportunities to cooperate, refer patients and discuss issues related to the patient group. This barrier was also related to the perceived uncertainty about which health care institution (and therefore which clinician) had chief responsibility for the patient. Two of the clinicians said:

*If a patient has had Alvedon for pain, for example, there’s absolutely no communication that this is what we prescribe, this is what we do, this is our bit. It’s only psychiatric. And primary care can take the other bit. And they think just the same, that no, psychiatry can prescribe that medication. So there’s absolutely no communication. And no one has main responsibility for the patient. If you’ve got a pain problem and are in touch with another clinic at the hospital there’s no contact between the doctors. (Clinician 4, psychiatric out-patient care).*

*The barriers are the shortage of doctors and lack of time with the doctor. Long waiting times, sometimes much too long so they have to go to the psychiatric emergency department instead. (Clinician 12 primary health care).*

## Facilitators

The results show that the main facilitators of access to somatic health care are the links between the patients with SMI and the medical departments. These take the form of functions (e.g. systems which ensure that patients receive regular reminders) or persons (e.g. professional contacts who facilitate patients’ access to health care). Other facilitators are clinical continuity and better knowledge about SMI patients.

### Individual-level facilitators

The individual-level facilitators of access to healthcare are the relatives who act as spokespersons for the patients and the clinicians’ own interest in improving their knowledge about the patient group and SMI.

The relatives perceived **themselves as spokespersons** who facilitated patients’ access to health care by accompanying them to appointments, asking questions and helping them with information about treatments, etc. The relatives described the spokesperson role in a positive way. However, those relatives who were parents of a son or daughter with SMI also discussed their worries about the future and who would support their child when they were no longer able to help. As one parent said:

*She doesn’t really understand that this (symptom, author’s note) isn’t good. That’s when we have to push as parents. And she’s got an appointment next week and then we can do our bit as parents… So of course we’ll go with her. But I think about if we haven’t got the strength or if we weren’t around, who would go with her then? Then there wouldn’t be any appointment. That’s when there would be consequences (Relative 12).*

The clinicians regarded their **own interest in developing a better understanding** of patients with SMI as a facilitating factor. A greater awareness of patients’ needs facilitated access to health care. Some of the clinicians had previous experience of psychiatric in-patient care and could therefore draw in this experience when caring for patients with SMI in primary or specialized care. However, bridging the knowledge gap was the responsibility of the individual clinician. Some of them reported that they, for example, searched for information on the Internet about patients’ symptoms and how to meet the needs of this patient group in an appropriate way.

### Organizational-level facilitators

The organizational-level factors which facilitated access to health care were seen to be: having a professional health-care contact; continuity among clinicians; and patients being contacted annually.

The patient’s **professional contact,** i.e. a nurse or assistant nurse at a psychiatric out-patient unit, was described by patients, relatives and clinicians as facilitating access to health care. The professional contact facilitated access by coordinating communication between the patient and the various health care units about such issues as medication, referrals and visits outside the psychiatric sector. Having a well-functioning professional contact was also seen as enhancing the patient’s trust in the health care system. One positive consequence of this was better adherence to referrals. As one patient commented:

*So, even though I do want them to know, in one way I also don’t want them to know. Because it’s a bit embarrassing. And I don’t know if they’ll get it, if they find out. It’s, for example, at my psychiatric clinic, they wanted me to start collecting my medication at the health centre instead. They wondered if I would be prepared to do it. No, I’m not going to get that medication there, I don’t want to because they don’t know anything about this kind of thing. And if I’ve got any questions or if I maybe refuse to take the medication… they won’t be able to help me with it. (Patient 2).*

**Continuity among clinicians** was reported to be an organizational-level facilitator of access to health care. Continuity facilitated exchange of knowledge between clinicians as well as continuity for the patients. Both patients and clinicians described continuity as an opportunity to build a patient-clinician relationship based on a trusting alliance. They described this as a vital factor in helping to prevent difficulties arising in patient contacts.

*Continuity of staff is vital, it’s absolutely vital. And especially for the weaker members of society, continuity is incredibly important for them. It’s more important than anything else, actually, and the health centre which has had the same staff for a long time, where there’s good continuity, well it obviously works much better, and when the doctor has known the patients since way back, then there’ll be fewer problems, so continuity is incredibly important. (Clinician 14).*

Both patients and clinicians regarded **annual reminders** about primary health care and psychiatric out-patient appointments as an organizational-level facilitator. The clinicians reported that the annual health surveys for patients in psychiatric care helped them to be better informed and facilitated contact.

## Discussion

The study’s aim was to explore the experiences and views of patients, relatives and clinicians about individual and organizational factors which either facilitate or hinder access to somatic health care for persons suffering from SMI. The results reveal the main barrier to be the gap between the organization of the health care system and the patients’ health care needs. Thus, the health care system is unable to meet the needs of patients with SMI. In addition, all patients reported self-stigma as a barrier to accessing health care. The main facilitators are the various links between SMI patients and the medical departments involved in their care. These facilitators can be functions, persons or factors such as clinical continuity and understanding of the SMI patients.

Primary care and general practitioners constitute the basic level of the Swedish health care system, both for somatic and psychiatric disorders. Patients with SMI are cared for by specialized psychiatric care services. Psychiatric out-patient units are mainly community based. They are thus located far from hospitals and seldom close to primary care centers. There is thus a geographical gap within the health care system that can be difficult to bridge for patients with SMI. There is also a knowledge gap within the system, in the sense that psychiatrists seldom know how to treat somatic disorders and vice versa. This is a consequence of the single-disease paradigm [21]. Specialist physicians in somatic health are often experts in a relatively small number of diseases, which means that a psychiatric patient with, for instance, type 1 diabetes and congestive heart failure, will need two somatic physicians as well as their psychiatrist. Patients with SMI and somatic comorbidities will thus be in contact with a number of medical departments. This can be a problem for any patient but is especially so for patients with impaired cognitive function and possibly also delusions. This is also the case for other patients with multi-morbidity such as the elderly [22]. It is therefore important for somatic clinicians to learn how to interact with SMI patients. As discussed below, physicians trained to provide somatic care in psychiatric settings might help to close or bridge this gap. We believe that this knowledge gap problem is an issue concerning all health care professionals.

Self-stigmatization was identified as an individual-level barrier for access to health care and was reported by all patients included in the study. This is in line with previous research [23–25]. Self-stigmatization is defined as a process in which the individual endorses stereotypes, avoids social interaction and regards him−/herself as a devalued member of society [23, 26]. Previous research has shown that self-stigma is negatively correlated with hope, self-esteem, empowerment and adherence to treatment [24, 25]. Self-stigmatization must be taken into account when developing interventions aimed at increasing access to health care for patients with SMI.

There is limited evidence about the effectiveness of interventions which aim to increase access to somatic health care for patients with psychiatric-somatic multi-morbidity [27]. A recent British report from the Royal College of Psychiatrists looks at the increased somatic disease burden among patients with SMI. The report suggests that a new specialist medical field of liaison physicians should be introduced [28]. One of the reasons for this is the geographical separation of somatic and psychiatric care that seems to be the rule in modern health care. As a consequence it is generally difficult for psychiatrists and their patients to consult physicians from somatic specialist disciplines. Patients tend instead to be referred to hospitals or primary care centers. This is often a problem for patients with cognitive impairments and leads to unnecessary delays or cancelled appointments, and ultimately impaired somatic health. A liaison physician is a general practitioner or specialist in internal medicine trained in the somatic comorbidities of severe mental illness. The liaison physician supports psychiatric departments and out-patient units both at individual patient level and at organizational level, for example treating patients for somatic comorbidities and teaching psychiatric staff about somatic diseases. The liaison physician could help to bridge the gap between somatic and psychiatric care, thus making it easier to meet the medical needs of psychiatric patients (cf. [27]). This recommendation is well in line with the results of the present study, which suggest that there should be professional contact personnel to act as links between SMI patients and the somatic health care services.

The present study highlights the potential of relatives and professional contact personnel to liaise between patients with SMI and the somatic health care services. This would help SMI patients to attend somatic medical appointments, overcome mistrust and maintain communication. However, the use of relatives and/or professional contacts has both advantages and disadvantages. On the one hand, our results showed that relatives regarded themselves as helping to empower patients to participate more actively in their own health care needs. This is in line with one systematic review, which demonstrated that knowledge provision and the patient’s perceived capacity are two key factors for successful shared decision-making [29]. On the other hand, the presence of relatives and professional contacts resulted in transparency between the persons involved, which in turn suggests a paternalistic perspective which may interfere with the patient’s legal right to autonomy. This was highlighted by the SMI patients in the present study. We therefore need to understand whether and in what way, the need for access to somatic healthcare for SMI patients may justify increased transparency.

One way to balance paternalism and the patient’s right to autonomy is to empower patients to share decision-making [29]. When this is the case, increased transparency can be seen as an empowering factor for patients with SMI. A study of conditions that help to create a good life for people with bipolar disorder demonstrates that professional and reliable private relationships can help individuals to carry out their wishes at times when they are not able to do so on their own [30]. Voluntary chosen dependency of this kind contributes to the quality of life of persons with bipolar disorder, while at the same time enhancing their power and control.

### Methodological considerations

The strength of the present study is that the data reflects a variety of perspectives (i.e. those of patients, relatives and clinicians). The participants (patients, relatives and clinicians) interviewed for the present study were strategically chosen to produce a wide variety of participants’ experiences. Of the 23 clinics which were invited to take part (primary and specialized somatic in-patient care), 13 declined due to heavy work load and limited time, even if their managers were willing to allow their clinicians to participate during working hours. We sought to include patients with a wide range of psychiatric diagnoses because patients with SMI are not a homogeneous group. Four of the patients who had agreed to participate withdrew their consent before the start of the interviews without giving any specific reason. The interview topics could have been perceived as violating personal integrity or recalling memories of feeling discriminated against. Two of the relatives withdrew from the study; none of the clinicians withdrew.

Another strength of the study was that the data collection was based on questions recommended by CFIR, aimed at identifying factors that hinder or facilitate access to health care for SMI patients. To ensure the integrity of the qualitative analysis and the results, the meaning units and codes were kept close to the interview data. Throughout the analysis process the researchers went back and forth between the interviews, meaning units and codes in several steps. In addition, the analysis was conceptually created and developed based on the data, and the results were continuously discussed in the inter-professional research group in order to improve rigour [18]. With regards to thematic saturation, we suggest that the data facilitated the categorization and abstraction of the results. Thus, saturation of the analysis was achieved [31].

The recruitment of patients by health care managers in primary and specialized health care may have affected the patients’ willingness to participate. However, we considered it necessary to use the recruitment process described here in order to create a safe setting for this vulnerable group of patients. Recruitment via managers gave patients the opportunity to discuss the interviews with their health care professionals. Because managers outside the research team were in charge of the recruitment process, it is not possible to give the exact number of non-participants. A relative limitation of the study is the small number of men it contains. In all other ways the strategic sampling was satisfactory.

## Conclusions

We conclude that health care services for SMI patients would benefit from reorganization. We recommend that structures and systems that facilitate cooperation between medical departments are established at organizational level. This might include economic incentives for cooperation and clinical continuity. Furthermore, we see the need for the ongoing education of professionals about SMI patients and somatic diseases which would include both medical knowledge and how to interact with SMI patients. We suggest that the links between individual and organizational level should be strengthened by the introduction of professional contact personnel in the form of liaison physicians and case managers who have a good knowledge of somatic conditions. This can reduce the levels of stress and responsibility experienced by relatives. The present study has a qualitative design which reflects the health care system in Sweden. We conclude that our results could also be of interest in other countries (with comparable health care systems) which are seeking to facilitate SMI patients’ access to somatic health care.

## Additional file

Additional file 1: Interview guides for semi-structured interviews with patients, relatives and clinicians. (DOCX 19 kb)

## Abbreviations

CFIR: Consolidated framework for implementation research
SMI: Severe mental illness
