# Supplementary material for: Access to primary and specialized somatic health care for persons with severe mental illness: a qualitative study of perceived barriers and facilitators in Swedish health care
Source: BMC Fam Pract. 2018 Jan 9;19:12. doi: 10.1186/s12875-017-0687-0 (PMC5759233; doi:10.1186/s12875-017-0687-0)
Supplement: Additional file 1: — Interview guides for semi-structured interviews with patients, relatives and clinicians. (DOCX 19 kb) [file 12875_2017_687_MOESM1_ESM.docx]

# Additional file 1

## Interview guides for semi-structured interviews with patients, relatives and clinicians

## Patients

Can you tell me about when you had to contact the health services because of a physical illness?

What obstacles/difficulties do you face when you need to interact with healthcare services?

Did the physician/nurse you met ask about your experience of mental illness?

How well do you think professionals you encounter in physical care show understanding for and knowledge about your needs?

Based on your experience, what simplifies your interactions with healthcare?

What makes your interactions with healthcare more difficult?

How might healthcare proceed to better meet your needs?

What changes to healthcare are needed, in your opinion, to better meet your needs?

## Relatives

Can you tell me about a situation in which your relative with severe mental illness had to contact the health services because of a physical illness?

How do you consider the care currently being provided to your relative?

How do you consider the competence of the healthcare services to meet your relative's needs?

How competent do you think healthcare (primary care, inpatient care) is to meet the physical care needs of individuals with severe mental illness?

What obstacles do you see in healthcare (primary care, inpatient care) to meeting physical care needs of individuals with severe mental illness?

How has healthcare competence affected the care provided to your relative when he or she has sought medical help for physical care needs?

What changes to healthcare are needed, in your opinion, to better meet your relative's physical healthcare needs?

## Clinicians

Can you tell me about how your unit meets the physical healthcare needs of patients with severe mental illness?

How do you view the care currently provided to individuals with severe mental illness who visit your unit seeking help for physical illness?

How is physical illness handled in psychiatry? Please give examples.

What level of competence do you think your unit has to meet the physical care needs of individuals with severe mental illness?

What obstacles do you see in your unit to meeting the physical care needs of individuals with severe mental illness?

What level of competence do you see in your organization for meeting the physical care needs of individuals with severe mental illness?

What obstacles do you see in your organization ("by and large") to meeting the physical care needs of individuals with severe mental illness?

Do you have guidelines or recommendations for working with the physical care needs of individuals with severe mental illness? If so, describe them.

What kind of support do you get from your immediate supervisor for your work with individuals with severe mental illness? What kind of support would you like to have?

How does healthcare competence affect the care provided to individuals with severe mental illness who seek care for physical care needs?

What competences are essential for adequate provision of care to individuals with severe mental illness who have physical care needs (e.g., support from one's supervisor, knowledge, professional networks)?

What changes to healthcare are needed, in your opinion, to better receive individuals with severe mental illness and to better meet their care needs??

How would you describe the needs of individuals with severe mental illness and physical care needs?

Do you think that your unit could improve the care it provides to individuals with severe mental illness? If so, how might this be done?
